# Effect of Propofol Versus Sevoflurane on Erections during Narcosis in Transurethral Surgery: The PENIS Trial

**DOI:** 10.1016/j.euros.2024.08.021

**Published:** 2024-09-24

**Authors:** Thomas P. Scherer, Corinna von Deschwanden, Ulrike Held, Cédric Poyet, Jia-Lun Kwok, Lukas Kandler, Martin Schläpfer, Etienne X. Keller, Florian A. Schmid

**Affiliations:** a Department of Urology, University Hospital Zurich, University of Zurich, Zurich, Switzerland; b Department of Anesthesiology, University Hospital Zurich, Zürich, Switzerland; c Department of Biostatistics, Epidemiology, Biostatistics and Prevention Institute, University of Zurich, Zurich, Switzerland; d Department of Urology, City Hospital Triemli of Zurich, Zurich, Switzerland; e Department of Urology, Tan Tock Seng Hospital, Singapore, Singapore

**Keywords:** Transurethral surgery, Penile tumescence, Intraoperative erection, Investigator-initiated clinical trial, Propofol, Sevoflurane

## Abstract

Penile erection is unwanted during transurethral interventions as it may be associated with adverse events such as impaired access, prolonged operation time, abortion of the procedure, or a need for ancillary measures to reach penis flaccidity, such as intracorporeal injection of vasoactive drugs. In recent years, anesthesia with propofol has been favored over sevoflurane for environmental reasons. To the best of our knowledge, there have been no prospective randomized clinical trials evaluating the impact of general narcosis medications on the risk of such unwanted penile erections during transurethral surgery. To fill this gap, we have planned a prospective, double-blind (surgeon and patient), single-center, randomized controlled trial. The primary outcome is the occurrence of an intraoperative penile erection. The secondary outcomes are related to the impact of the primary outcome on the surgery, such as changes in operative strategy or operation duration, abortion of the procedure, and adverse events. The plan is to randomize 200 patients undergoing transurethral surgery to receive general anesthesia with either propofol or sevoflurane. The inclusion criteria are men aged <75 yr with an International Index of Erectile Function-5 score of ≥12 points. All men fulfilling the inclusion criteria will be asked to participate. Exclusion criteria are patient characteristics associated with a higher risk of complications with the use of either propofol or sevoflurane. Randomization and treatment allocation will occur after patients give consent. The results will be statistically analyzed using a logistic regression model. This research has received ethical clearance from the local ethics committee (KEK code 2023-01682). The trial is registered on the Swiss National Clinical Trials Portal (SNCTP000005681) and on ClinicalTrial.gov (NCT06378645).

## Take Home Message

Unwanted penile erection can be problematic during transurethral surgery. There has been a recent move towards propofol anesthesia over sevoflurane for environmental reasons. To address the lack of robust clinical evidence regarding the effect of anesthesia agents on erection risk during transurethral surgery, we are planning a prospective study to compare the effects of propofol versus sevoflurane.

## 1 Introduction and hypotheses

Intraoperative penile erection is unwanted during transurethral surgery, since it may impair access to the urinary pathways thus prolonging the operation time and can sometimes require ancillary measures to reach penis flaccidity. Ultimately, penile erection may cause abortion of a procedure. Reversal of penile erection can be achieved intraoperatively via pharmacological interventions, including intravenous systemic administration of ketamine or local intracorporeal injection of a vasoactive agent such as phenylephrine or epinephrine. However, these medications are in turn associated with potentially severe secondary effects [1], [2], [3]. Incidence rates for intraoperative penile erection in adults range between 0.1% and 3.5% according to the literature [4], [5]. Evidence on the topic is of limited quality, and the risk factors for and frequency of undesired penile erections in transurethral interventions remain to be evaluated.

The use of propofol has been associated with a particularly high risk of sexual arousal and intraoperative erection, with an incidence of up to 10% in pediatric case series undergoing urethral surgery [6]. The causes and pathophysiology have been explored in parts, but the leading mechanisms remain a matter of debate [7], [8]. In an effort to reduce greenhouse gas emissions, primary use of propofol instead of gaseous agents for general anesthesia was introduced at our tertiary academic hospital in October 2022 [9]. This institutional change in anesthesia protocol has led to an increase in the rate of unwanted intraoperative erections and interventions to achieve penile flaccidity, potentially associated with the increase in the use of propofol. Because there are no high-quality studies in the literature on the impact of various anesthetic agents on penile tumescence in men during transurethral procedures, anesthesiologists and urologists at our institution initiated a prospective randomized controlled trial (RCT) comparing the incidence of intraoperative penile tumescence during propofol versus sevoflurane general anesthesia.

## 2 Study design

The PENIS trial is a single-center, double-blind, superiority RCT. The superiority of a drug in this context is defined as the one that causes fewer erections during transurethral surgery. The study will be conducted at the Department of Urology, University Hospital Zurich, Zurich, Switzerland. The trial started in February 2024. It is estimated that recruitment will end in December 2026.

### 2.1 Endpoints

The primary endpoint of penile erection will be analyzed as a binary outcome (flaccid or erect). The occurrence of penile tumescence at any time during surgery is classified as an erect penis. Conversely, a flaccid penis is classified only in the absence of penile tumescence at any point during the surgery. The start and end of the operation are defined as insertion and retrieval of transurethral instruments, respectively. Penile tumescence will be reported using a simplified version of the validated Erection Hardness Score [10], where 0 = flaccid, 1 = increased tumescence, but not fully erect, and 2 = full erection. Erect penile tumescence is defined as a score of either 1 or 2, whereas a flaccid penis is defined as a score of 0.

Secondary outcome measures will evaluate whether penile tumescence leads to any of the following:

1. Subjective prolongation of the intervention (time in minutes).
2. Change in the operative strategy (any change counts).
3. Adaptation of instruments and approaches (any adaptation counts).
4. Change to the operative outcome (any subjective change in operative outcome counts).
5. Necessity for drug application to decrease penile tumescence (intravenous ketamine will be used as the first-line treatment; if ketamine is unsuccessful, intracorporal phenylephrine will be administered; either of these two drugs or the combination thereof counts).
6. Complications that could plausibly be related in time to intraoperative penile tumescence and cannot be explained otherwise (any complication counts and will be classified according to the Clavien-Dindo scheme).

### 2.2 Eligibility criteria

The inclusion criteria for the trial are defined as follows:

Men scheduled for transurethral surgery at the Department of Urology, University Hospital Zurich;
Age 18–75 yr;
Preoperative International Index of Erectile Function 5-item (IIEF-5) score of ≥12 points; and
Expected operation time ≥15 min.

Contraindications or allergy to propofol or sevoflurane will lead to exclusion of the patient.

### 2.3 Interventions

The participants will receive general anesthesia based on either propofol (intervention) or sevoflurane (control). Propofol will be administered intravenously using the target-controlled infusion algorithm (Schnider model) with effect-site concentrations of 1.5–4.0 μg/ml necessary. Sevoflurane will be given in gaseous form via an endotracheal tube at concentrations of 1.5–2.5% by volume. Both study medications will be applied by the anesthesiologist. In this study, both study groups will receive the authorized medication according to an internal standard operating procedure protocol (Table 1).

**Table 1:** Anesthesia protocol and supportive medications, including management strategies for intraoperative erections, for the propofol and sevoflurane groups

	Sevoflurane group	Propofol group
Airway management	Intubation	Intubation
Medications	Thiopental 5 mg/kg	Fentanyl/remifentanil TCI
	Fentanyl/remifentanil TCI	Propofol TCI (Schnider model)
	Sevoflurane	Rocuronium bromide
	Rocuronium bromide	
Supportive medications	Ephedrine	Ephedrine
	Norepinephrine	Norepinephrine
	Dexamethasone	Dexamethasone
	Ondansetron	Ondansetron
In case of an erection		
First line	Ketamine 0.5 mg/kg	Ketamine 0.5 mg/kg
Second line	Phenylephrine intracorporally	Phenylephrine intracorporally

TCI = target-controlled infusion.

### 2.4 Sample size determination

On the basis of the literature outlined in Section 1, we expect the incidence of penile erection to be approximately 1% among patients receiving sevoflurane-based anesthesia and approximately 10% among those receiving propofol. To detect this 9-percentage-point difference in risk, which we consider a clinically relevant difference, and to assure a study power of 80% with a dichotomous outcome variable and a two-sided significance level of α = 0.05, 100 patients will be needed in each group (200 patients in total). The sample size calculated is appropriate to the hypothesis being tested, so that any results will be appropriately generalizable.

### 2.5 Enrolment and study schedule

**Fig. 1:**
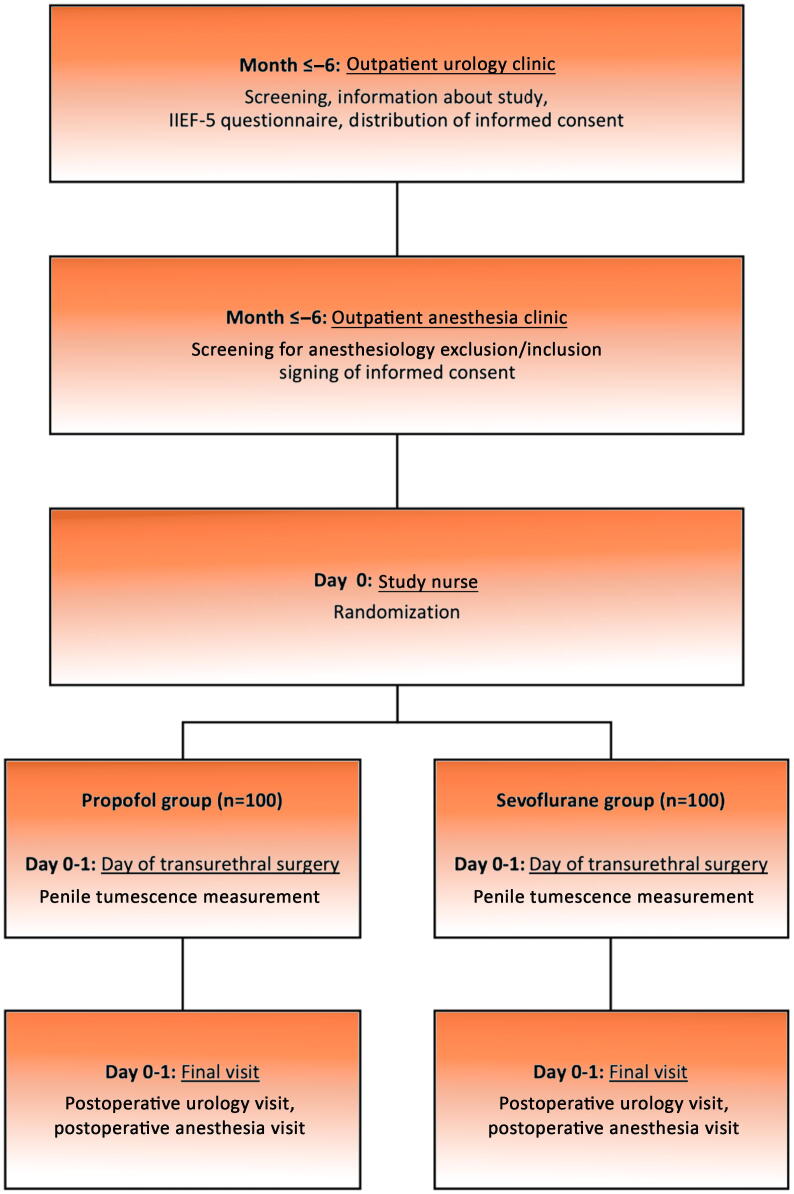
Flow diagram illustrating the time schedule for patient activities, interventions, and control therapies, as well as measurement of primary and secondary endpoints throughout the study period.

Every male patient referred for surgical consultation before transurethral surgery will be screened by age and IIEF-5 score. If the patient is eligible for participation in the study, he will be informed accordingly. After giving informed consent, patients will be randomized to the intervention or control group via computerized random sequence generation on the day of surgery. The study flow chart is shown in Figure 1. Within 1 d after the intervention, patients will be visited by the urology and anesthesiology team for end-of-study assessment.

### 2.6 Assignment of interventions and allocation concealment

The randomization list will be prepared in the R statistical software environment [11]. We will use random block sizes to maintain allocation concealment, as well as equally sized groups throughout the course of the trial to minimize the risk of bias. The IIEF-5 score (dichotomized at ≥22 points = no erectile dysfunction vs ≥12 to ≤21 points = mild or mild-moderate erectile dysfunction) will be used for stratification. The randomization list will be prepared by the trial statistician. Audits by an independent person will oversee the preparation of the randomization list and assure the monitoring of the study. Sealed envelopes will be filled according to the randomization list to guarantee allocation concealment. During the recruitment process, we will sequentially assign each participant the next available envelope in the randomized sequence.

### 2.7 Data collection

The following parameters will be recorded for each patient: age, blood pressure, heart rate, weight, height, IIEF-5 score, as well as the reason for the type and duration of the transurethral intervention. All primary and secondary outcomes will be reported immediately before operation sign-out by the operating urologist in the presence of the anesthesiologist. Until the end of the study visit, all patients will be screened for related adverse events. In the case of an adverse event, the type, grade, and time until resolution will be recorded. All parameters are entered into a secure electronic database.

### 2.8 Blinding

The authorized study nurse will hand the upcoming envelope with the treatment allocation to the anesthesiologist. The patient and the urologist (who is measuring the outcome) will not know which form of general anesthesia will be administered. To this end, blankets in the operating room will hide installations used by the anesthesiologists to prevent the urologist witnessing the assigned narcosis form. Before surgical sign-out, while still scrubbed in and with the patient under anesthesia, the anesthesiologist will ask the urologist about the outcome questionnaire. Together, they will fill in the paper case report form (CRF).

### 2.9 Auditing, data monitoring, and harms

Monitoring visits will be performed before the start and during the study period at the investigator’s site. During internal audits, all paper CRFs and the electronic database will be inspected. Since both anesthesiological drugs are licensed for use, a continuous data monitoring board will not be necessary and no interim analysis is planned, in accordance with local laws and ethical principles. Any complication associated with the investigational drugs will be recorded as a secondary outcome, as detailed above.

### 2.10 Ethical considerations

The protocol, the proposed participant information and consent form, and other study-specific documents were approved by the local ethics committee in compliance with legal requirements (KEK-Nr. Code 2023-01682). The study will strictly follow the protocol. If any changes become necessary, an amendment to the protocol will be formulated.

### 2.11 Statistical methods

Before data export, a statistical analysis plan will be written up and signed by the principle investigator and the trial statistician. The analysis will be performed blinded and using dynamic reporting tools in a fully scripted way to guarantee highest standards regarding reproducibility. The primary analysis of the primary outcome will be performed using a logistic regression model with a logit link function to the treatment group and the stratification variable as an independent variable. The effect measure will be an odds ratio.

### 2.12 Consent

All patients will be informed about the study by trained urologists and anesthesiologists. Inclusion in the study will only occur after written consent has been obtained from the patient. When a participant withdraws consent, data collected before the date of withdrawal remain within the electronic study database and can be included in data analysis. Participants who discontinue the study before data collection will be replaced. Individual medical information obtained within this study is considered confidential, and disclosure to third parties is prohibited. Subject confidentiality will be further ensured by using patient identification code to correspond to treatment data in the computer files. For source data verification purposes, authorized representatives of the investigator, a competent authority, or monitoring body may require direct access to parts of the medical records relevant to the study, including participants’ medical history. The planned clinical trial entails only minimal risks for study participants, considering that both of the investigational drugs are licensed for use by the Swiss medical authorities and have been regularly used in daily routine for many years.

### 2.13 Dissemination

After statistical analysis of this trial has been performed, the sponsor and investigator will publish the data in a peer-reviewed medical journal. Before publication, the findings will be presented at appropriate congresses. Authorship will be based on the following criteria: an author must significantly contribute to the study conception or design, participate in drafting or critically revising the content, approve the final version for publication, and be accountable for the accuracy and integrity of the work. Contributors who do not meet all four criteria will be listed in the acknowledgments section. After successful publication, the data will be made available in anonymized form in an open-access repository.

## 3 Summary

This prospective RCT is comparing the incidence of penile erection during transurethral interventions under general anesthesia with either propofol or sevoflurane. Given the global shift towards reducing greenhouse gas emissions and the consequent decrease in the use of gaseous anesthetics, the study addresses a potentially critical yet underexplored topic in the context of transurethral surgeries [9].

Should our study confirm the hypothesis that either propofol or sevoflurane is associated with significantly higher rates of intraoperative erections, this finding may have an impact on anesthesia protocols for transurethral surgeries. Should the study find a significantly higher erection rate with propofol, then urology and anesthesiology departments would face the ethical and clinical dilemma of balancing the environmental benefits of propofol against the greater risk of complications and prolonged operation times during transurethral surgeries. Conversely, should sevoflurane be associated with a significantly higher rate of erections, the rationale for the use of propofol instead of gaseous anesthetics would be strengthened. Furthermore, such a result would highlight the need for a better understanding of the pathophysiological mechanisms underlying intraoperative erections and their management. Hence, this study will not only fill a significant gap in the medical literature but should also serve as a catalyst for further discussion on the ecological impact of health care.

  ***Author contributions:*** Thomas P. Scherer had full access to all the data in the study protocol and takes responsibility for the integrity of the data and the accuracy of the data analysis.

  *Study concept and design*: Scherer, von Deschwanden, Poyet, Schläpfer, Keller, Schmid, Held.

*Acquisition of data*: Scherer, von Deschwanden, Held, Kandler, Keller, Schmid.

*Analysis and interpretation of data*: Held.

*Drafting of the manuscript*: Scherer, Schmid, Held, Kwok.

*Critical revision of the manuscript for important intellectual content*: von Deschwanden, Poyet, Kandler, Schläpfer, Keller.

*Statistical analysis*: Held.

*Obtaining funding*: Keller.

*Administrative, technical, or material support*: von Deschwanden, Held, Schläpfer, Schmid, Keller.

*Supervision*: Poyet, Schläpfer, Keller, Schmid.

*Other*: None.

  ***Financial disclosures:*** Thomas P. Scherer certifies that all conflicts of interest, including specific financial interests and relationships and affiliations relevant to the subject matter or materials discussed in the manuscript (eg, employment/affiliation, grants or funding, consultancies, honoraria, stock ownership or options, expert testimony, royalties, or patents filed, received, or pending), are the following: None.

  ***Funding/Support and role of the sponsor:*** The study is financed by an internal University Hospital Zurich innovation grant (INOV00069) guaranteeing involvement of a study urology nurse (20%). The sponsor will play a role in management of the data.

  ***Ethics considerations:*** The protocol, proposed participant information and consent form, and other study-specific documents were approved by the local ethics committee in compliance with legal requirements. The local ethics board is Cantonal Ethics Committee Zurich (Stampfenbachstrasse 121, CH 8090 Zürich, Switzerland; Tel. +41 43 2597970; E-mail address: info.kek@kek.zh.ch). The approval reference number is 2023-01682. Each patient gives written consent before study inclusion.

  ***Data sharing statement:*** After publication, the data will be made available in anonymized form in an open-access repository.
